# Limited role of functional differentiation in early diversification of animals

**DOI:** 10.1038/ncomms7455

**Published:** 2015-03-04

**Authors:** M.L. Knope, N.A. Heim, L.O. Frishkoff, J.L. Payne

**Affiliations:** 1 Department of Geological & Environmental Sciences, Stanford University, 450 Serra Mall, Building 320, Stanford, California 94305, USA; 2 Department of Biology, Stanford University, Gilbert Hall, Stanford, California 94305, USA

## Abstract

The origin of most animal phyla and classes during the Cambrian explosion has been hypothesized to represent an ‘early burst’ of evolutionary exploration of functional ecological possibilities. However, the ecological history of marine animals has yet to be fully quantified, preventing an assessment of the early-burst model for functional ecology. Here we use ecological assignments for 18,621 marine animal genera to assess the relative timing of functional differentiation versus taxonomic diversification from the Cambrian to the present day. We find that functional diversity increased more slowly than would be expected given the history of taxonomic diversity. Contrary to previous inferences of rapid ecological differentiation from the early appearances of all well-fossilized phyla and classes, explicit coding of functional characteristics demonstrates that Cambrian genera occupied comparatively few modes of life. Functional diversity increased in the Ordovician and, especially, during the recoveries from the end-Permian and end-Cretaceous mass extinctions. Permanent shifts in the relationship between functional and taxonomic diversity following the era-bounding extinctions indicates a critical role for these biotic crises in coupling taxonomic and functional diversity.

## Abstract (web summary)

Functional differentiation and taxonomic diversity are related in modern ecosystems. Here, the authors show that functional differentiation lags behind taxonomic diversification early in the evolutionary history of marine animals and that important shifts in this relationship occur at major mass extinction events.

## Graphical abstract

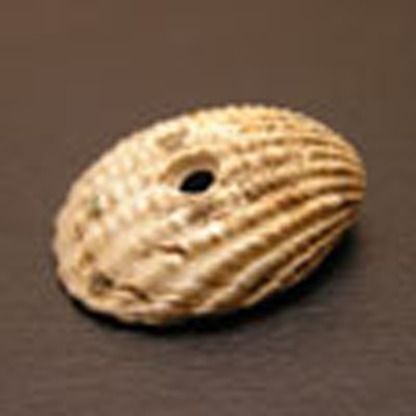

Functional differentiation plays an important role in supporting taxonomic diversity in modern ecosystems^1^,^2^,^3^,^4^ and has also long been hypothesized to account for the increase in animal taxonomic diversity across geological time^5^,^6^, but the hypothesized links between taxonomic and functional diversity over evolutionary time have never been tested quantitatively. Whereas the taxonomic diversity history of marine animals has been known in broad form for more than 150 years^7^ and in increasing detail over the past several decades^8^,^9^,^10^, the complete functional history of marine animals remains largely unknown^11^. Early work demonstrated that functional diversity increased throughout the Phanerozoic^12^,^13^ and explicit ecological categorization of selected assemblages from modern oceans and the Ediacaran through Ordovician periods (635–444 Myr ago) confirmed that the number of ecological modes of life within marine communities increased at some point during the past 444 million years^14^,^15^,^16^. However, these studies lack the taxonomic and temporal coverage to assess quantitatively the timing of taxonomic diversification versus ecological differentiation.

Studies of morphological disparity in the fossil record, a potential proxy for ecological differentiation^5^, generally support an ‘early-burst’ scenario in which morphological disparity was quickly maximized during the Cambrian explosion and subsequent taxonomic diversity has mostly evolved within those fundamental body plans^17^,^18^,^19^,^20^,^21^. However, it is unclear if the early-burst model of evolution applies also to ecological modes of life because there are many examples of functional overlap across disparate body plans. For example, tunas and squid are both fully motile, pelagic predators and adult oysters and barnacles are both non-motile, suspension feeders living on the sediment–water interface.

The relationship between ecological differentiation and taxonomic diversification can potentially fall into one of three simplified scenarios: (i) an ‘early burst’, (ii) a ‘time-constant’ and (iii) a ‘late-filling’ scenario. Under an early-burst scenario, diversifying animals quickly fill most basic functional ecologies in the beginning of the Phanerozoic even though taxonomic diversity is low, a pattern generally consistent with morphometric data^17^,^18^,^19^,^20^,^21^. In this scenario, later diversification occurs differentially within already-occupied modes of life and therefore rarefaction curves describing the number of ecological modes of life versus genus diversity should decrease in slope over time, as a given subsample of taxa will occupy fewer ecological modes as taxonomic diversity outpaces the evolution of new ecological modes (Fig. 1a). Under a time-constant scenario, the rarefaction curve describing the number of ecological modes relative to taxonomic diversity does not change over time (Fig. 1b). In this scenario, earlier intervals are equivalent to subsamples of later intervals. The total number of ecological modes occupied will increase over time with taxonomic diversity, but the fundamental distribution of genera among modes of life will remain unchanged and so all intervals will fall along the same rarefaction trajectory. Importantly, the ratio of genera to ecological modes changes with taxonomic diversity even under the time-constant model (Fig. 1b) and so previous assessments of taxonomic and functional diversity that did not include explicit assignments for all genera cannot be used to test against this null model. Under a late-filling scenario, ecological modes are progressively added across long expanses of geologic time and subsequently filled with taxonomic diversity. In this scenario, the rarefaction curves increase in slope over time because genera become progressively more evenly distributed among modes of life (Fig. 1c).

We assigned each genus of marine animal to an ecological mode of life^15^,^16^ (hereafter ‘ecological mode’) using the first principles of functional morphology. Assignments for tiering position relative to the sea floor, motility level and feeding strategy were taken from the literature (see Supplementary Data 1 for individual references). Assignments for the combination of these three ecological axes constitute the ecological mode of the genus (see Supplementary Fig. 1 for all ecological modes filled across the Phanerozoic). In total, we assigned an ecological mode to 18,621 marine animal genera that also have geologic stage-resolved (the most finely separated intervals of global geologic time for global genus diversity data) first and last appearances in the fossil record, creating a data set of 161,947 unique genus-by-stage combinations. This data set covers 63% of genera with stage-resolved stratigraphic ranges^22^,^23^,^24^ and spans the nine major marine animal phyla in the fossil record: Arthropoda, Brachiopoda, Bryozoa, Chordata, Cnidaria, Echinodermata, Hemichordata, Mollusca and Porifera.

In contrast to previous claims of an ‘early burst’^17^,^18^,^19^,^20^,^21^, we find that marine animals have followed a ‘late-filling’ pattern in which basic functional differences among animals have accumulated more slowly than would be expected given the history of genus diversity. Rather than being stimulated by functional innovation, the Cambrian explosion occurred despite strong functional similarities among distantly related genera. Moreover, Palaeozoic oceans show no significant correlation between the number of genera and the number of ecological modes within Linnaean classes. The increase towards modern functional diversity primarily occurred during the Ordovician and during recovery from the end-Permian and end-Cretaceous mass extinctions. Although taxonomic and functional diversity were largely decoupled during the early evolution of marine animals, post-Permian taxonomic diversity within Linnaean classes is predictable from early functional differentiation, suggesting functional diversity has played a key role in survival and recovery from the era-bounding mass extinction events.

## Results

### Ecological modes and genus diversity across time

**Figure 1:**
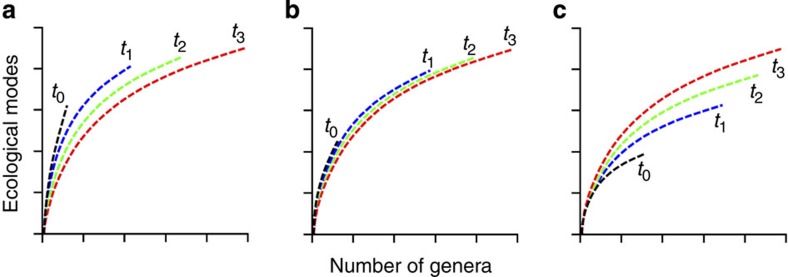
Hypothetical ecology–diversity relationships in the history of marine animal life. Conceptual diagrams showing hypothesized rarefaction curves describing the relationship between number of genera and number of ecological modes across the Phanerozoic (541 Myr ago to the present). (**a**) Early-burst scenario, where the number of ecological modes of life relative to taxonomic diversity is high early and the slope of the curve describing the number of ecological modes filled by equivalent subsamples of genus diversity becomes reduced with time; (**b**) time-constant scenario, where the number of ecological modes of life simply tracks taxonomic diversity across time; (**c**) late-filling scenario, where new ecological modes of life are progressively added across time and subsequently filled with taxonomic diversity.

**Figure 2:**
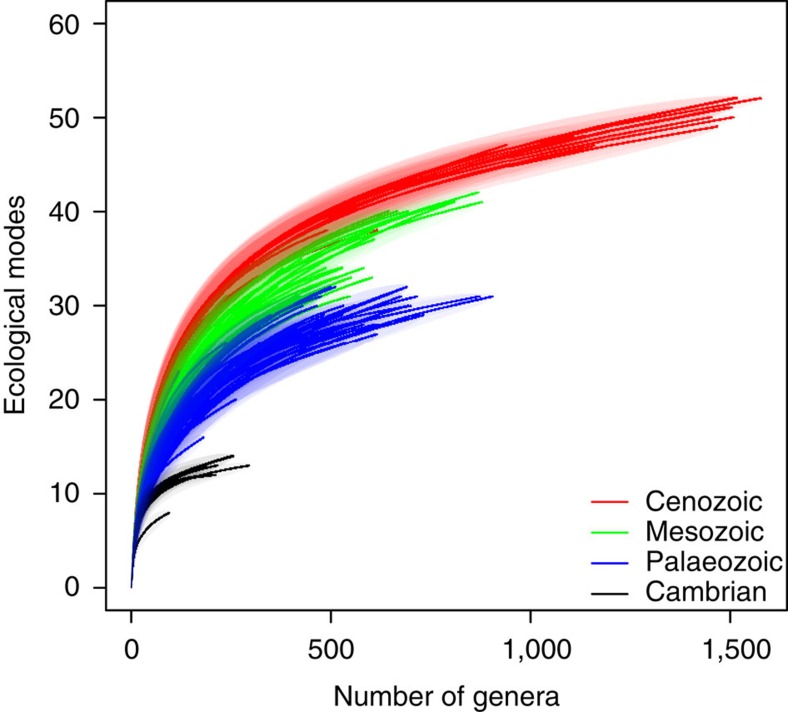
Ecology–diversity relationships of marine animals across the past 541 million years. Rarefaction curves of number of ecological modes in each geologic stage as a function of genus diversity. Each curve represents the mean rarefaction relationship for an individual geologic stage (± s.e. as shading). Geologic stages are coloured according to the era they belong to, with the Cambrian plotted separately from the rest of the Palaeozoic.

The accumulation of ecological modes best fits a late-filling scenario (compare Figs 1c and 2). The structure of the ecology–diversity relationship is clearly distinct in the Cambrian, during the rest of the Palaeozoic, throughout the Mesozoic and again throughout the Cenozoic, with persistent increase across eras in both total number of occupied ecological modes and the trajectories of rarefaction curves describing the functional versus taxonomic diversity relationship (Fig. 2), as previously hypothesized^12^,^13^. In contrast to the clear differences among eras, there is no systematic relationship between time and the functional versus taxonomic diversity relationship within eras: the post-Cambrian Palaeozoic shows no systematic relationship across geologic periods; Mesozoic rarefaction curves largely separate the Cretaceous from the Triassic, but the Jurassic overlaps both and Cenozoic rarefaction curves display similar ecology–diversity relationships across geologic periods (Supplementary Fig. 2).

### Comparison of actual history with a time-constant model

**Figure 3:**
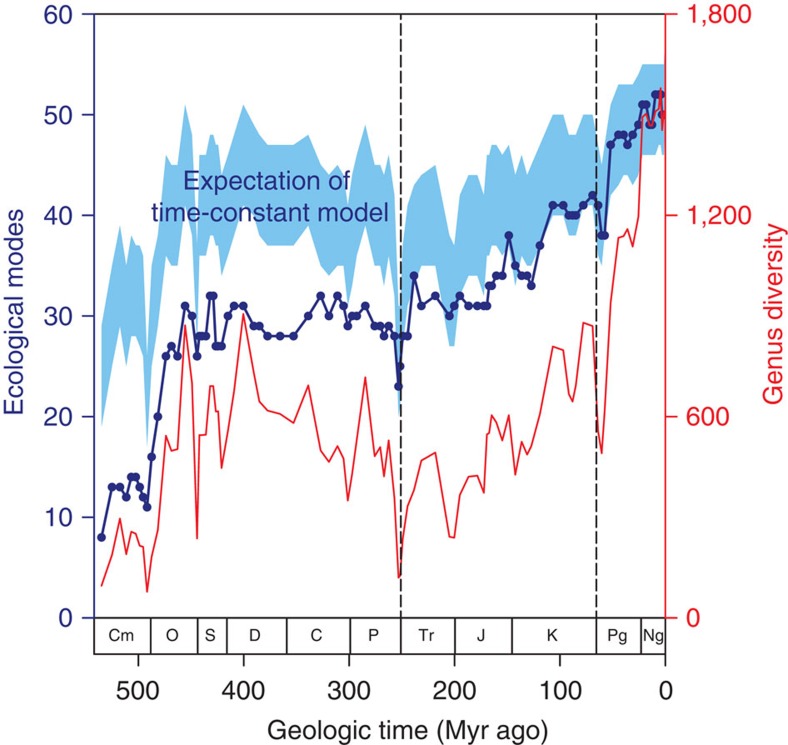
Changes in the number of ecological modes of life occupied by marine animals over the past 541 million years. Blue line with filled points depicts ecological modes; red line depicts genus diversity in our study with an assignment to an ecological mode; the blue polygon illustrates the 95% confidence interval for the expected number of ecological modes relative to observed genus diversity in each stage under the time-constant scenario, where the shape of the rarefaction curve describing the relationship between genera and modes of life remains constant across time. Dashed vertical lines demarcate the era-bounding mass extinctions at the P/Tr (252 Myr ago) and K/Pg (66 Myr ago) extinction horizons.

The Phanerozoic histories of total genus diversity and ecological modes occupied further illustrate the support for the late-filling model and indicate the role of mass extinctions in accelerating the exploration of ecological modes. The trend in the observed number of ecological modes from below the prediction of the time-constant model in the Cambrian (and the remainder of the Palaeozoic) to within the prediction of the time-constant model in the Cenozoic is likewise consistent with the late-filling model (Fig. 3; see also Supplementary Figs 8 and 9). This scenario would only be possible if the evolution of ecological novelty was slow to develop and not simply a function of standing taxonomic diversity during each geologic stage, let alone a prerequisite for diversification. Only after the Permian/Triassic (P/Tr) extinction at 252 Myr ago does the number of ecological modes begin to occupy the lower bounds of the expected value for the Phanerozoic. However, it is not until the recovery from the Cretaceous/Paleogene (K/Pg) extinction at 66 Myr ago that ecological modes permanently occupy the 95% confidence interval (Fig. 3). The other major mass extinction events (end-Ordovician, Late Devonian, end-Triassic) had comparatively minor effects on the number of ecological modes in the oceans and the most rapid rate of origination of ecological modes occurred in the Early Ordovician (488 Myr ago; Supplementary Fig. 3), coincident with the rapid diversification of the Palaeozoic Fauna^25^.

### The role of mass extinctions

The clear differences in the trajectories of the rarefaction curves among eras (Fig. 2) along with the lack of separation of rarefaction curves within eras (Supplementary Fig. 2) further indicates that the era-bounding mass extinctions have had a disproportionate influence on the relationship between taxonomic diversity and ecological differentiation. The era-bounding mass extinctions (P/Tr and K/Pg) caused some loss of ecological modes (loss of five modes at each; Fig. 3 and Supplementary Fig. 4), whereas recovery intervals ~20 Myr after the P/Tr and K/Pg mass extinctions both display high levels of origination of modes of life (14 new or recovered modes after P/T and 10 new or recovered modes after K/Pg; Fig. 3 and Supplementary Fig. 3), supporting the findings of recent work that suggests niche conservatism is weaker after mass extinctions^26^. Genus diversity within surviving ecological modes exceeded pre-extinction event levels within ~10 Myr of these extinction events and, in the case of the P/Tr, the number of occupied ecological modes already exceeded pre-extinction levels well before taxonomic diversity recovered (Fig. 3). Genera were preferentially lost from ecological modes that had high genus diversity before the P/Tr (ordinary least-squares linear regression of proportional change in taxonomic diversity within ecological modes to standing diversity before the extinction event: *N*=29 ecological modes both before and after extinction event, *R*^2^=0.22, *P*=0.01; see Methods for details), and K/Pg extinction events (linear regression: *N*=42 ecological modes both before and after extinction event, *R*^2^=0.11, *P*=0.03; see also Supplementary Fig. 4), thus permanently shifting the statistical relationship between ecological modes and genus diversity to higher values.

### Correlation between ecological modes and genus diversity

**Figure 4:**
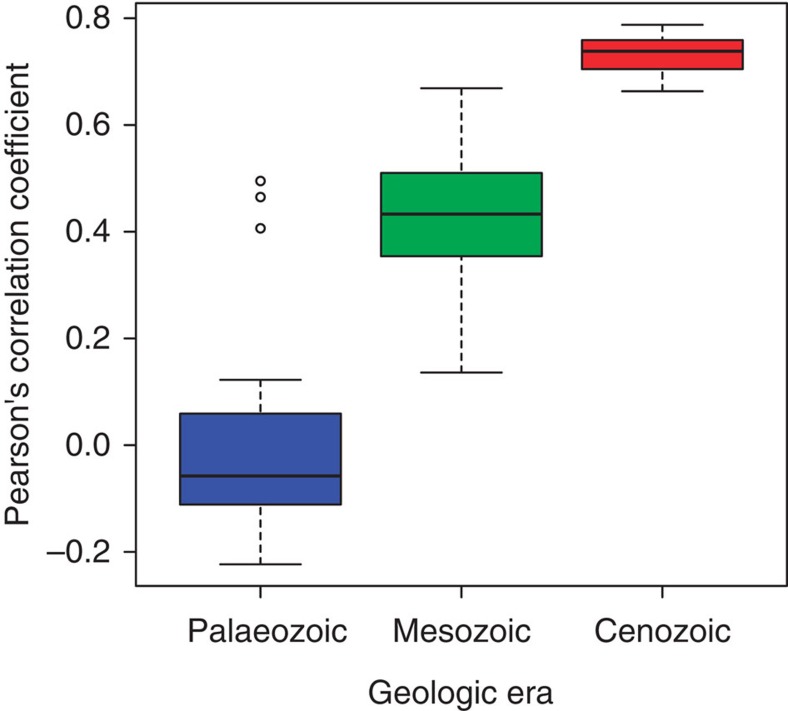
Box and whisker plots of the correlation between ecological modes and taxonomic diversity within Linnaean classes have increased steadily across geologic eras. Generalized least-squares model that includes the geologic eras as a covariate performs significantly better than a model with temporal autocorrelation alone (Likelihood ratio=60.5, df=2, *N*=89 stages, *P*<0.0001). The Cambrian is not included here because there were too few genera to calculate a per stage correlation coefficient during this period. The box represents the upper and lower quartiles and the dark line the median value. The whiskers extend to the minimum and maximum data point in cases where there are no outliers, which are indicated with open circles (Palaeozoic only).

In addition to the overall increases in the number of ecological modes occupied and the number of genera among all ecological modes, the correspondence between taxonomic diversity and number of ecological modes within Linnaean classes has also increased across the Phanerozoic (Fig. 4). During the Palaeozoic, taxonomic diversity within a given class was essentially uncorrelated with the number of ecological modes occupied by that class. In contrast, substantial correlation between ecological modes occupied and genus diversity developed early in the Mesozoic and increased in strength towards the present day (Fig. 4 and Supplementary Fig. 5).

Although functional and taxonomic diversity within classes were not significantly correlated during Palaeozoic time, future taxonomic diversification is predictable from early Palaeozoic ecological differentiation. For example, the number of ecological modes occupied by a Linnaean class at the end of the Ordovician radiation (Katian stage; 445 Myr ago) does not correlate with the genus diversity of that class at that time (linear regression: *N*=23 classes, *R*^2^=0.11, *P*=0.76). However, the number of ecological modes occupied during the Katian does predict the genus diversity of the class in the Pleistocene, 444 million years later (linear regression: *N*=33 classes in both Katian and Pleistocene, *R*^2^=0.56, *P*=0.003) and the total Phanerozoic taxonomic diversity of the class (linear regression: *N*=55 classes across all Phanerozoic time, *R*^2^=0.20, *P*=0.002). Although the end-Permian mass extinction was the proximal trigger of the shift from a world dominated by brachiopods and crinoids to one dominated by bivalves and gastropods^25^,^27^, the most taxonomically diverse classes in the modern marine fauna already had a higher propensity for functional ecological possibility that was in place far earlier.

## Discussion

Several factors may account for the discordance between morphological disparity, which supports an early-burst model^17^,^18^,^19^,^20^,^21^, and ecological modes, which best fit a late-filling model. High early body plan disparity amongst and within animal phyla may not have directly translated into high early ecological differentiation at the scale of ecological modes for at least three reasons: first, taxa can have vastly disparate body plans but the same basic functional ecology (for example, tunas and squid). Second, a single basic body plan can progressively give rise to many ecological modes without occupying new regions of overall animal morphospace (for example, gastropods, bivalves^28^). Third, high early morphological disparity in some groups^18^,^19^,^20^,^21^ may not directly translate to high early ecological differentiation, as much of the anatomical disparity (for example, number of trilobite body segments) would not have changed the fundamental ecological modes of these taxa.

Although the advent of macrophagous predation undoubtedly played a role in the Cambrian explosion^19^,^29^,^30^, the low functional diversity in Cambrian and Palaeozoic oceans and the general lack of correlation between taxonomic and functional diversity within classes suggests that they remained an ecological Garden of Eden. Taxonomic diversity, within and among Linnaean classes, did not depend on functional differentiation. We hypothesize that as functional diversity increased following the era-bounding mass extinctions, and especially the diversity and abundance of motile and predatory species climbed^15^,^31^, the number of ecological demands on species also increased. This increase in the number of competing needs would have roughened the evolutionary landscape^32^, causing increases in both taxonomic and functional diversity and thereby tightening the correspondence between the number of ecological modes and the number of genera. Regardless of the precise explanation, however, explicit coding of functional ecology reveals that while animal phyla exploded onto the scene during the Cambrian, the exploration of ecological possibility and the coupling of taxonomic to functional diversity has been a more protracted affair.

## Methods

### Diversity and age estimates

To avoid taxonomic uncertainties of the validity of subgenus designations, all subgenera were elevated to genus status, as is common practice in palaeontological research^10^. Our data set consists of 1,344 arthropod, 3,956 brachiopod, 379 bryozoan, 1,463 chordate, 1,790 cnidarian, 2,003 echinoderm, 253 hemichordate, 6,551 mollusc and 882 sponge genera. Stratigraphic ranges for genera were collected from the Treatise of Invertebrate Paleontology^23^, A Compendium of Fossil Marine Genera^24^ and the Handbuch der Paläozoologie [Handbook of Paleozoology]^22^. These synoptic sources were used rather than the Paleobiology Database for data on genus diversity and stratigraphic range because the Paleobiology Database is not sufficiently complete at this taxonomic scale. For example, it contains stage-resolved occurrences for only 17% of chordate genera, 49% of molluscs, 50% of echinoderms and 56% of arthropods and brachiopods in our data set. Only genera with stage level resolution of first and last appearance dates in the fossil record were included. If a genus had conflicting stratigraphic range information in any of these sources, the most recently published reference was used. The Geological Society of America’s v. 4.0 timescale^33^ was used for age boundaries of geologic intervals. Data are plotted in all cases at the midpoint of geologic stages.

### Sampling bias

The first-order patterns in this data set are not likely to be strongly influenced by sampling bias for the following reasons. The nine phyla included in this study comprise all of the marine animals with readily fossilizable elements^24^. The 18,621 genera included in this study have stage-resolved stratigraphic ranges in the fossil record and comprise 63% of the genus diversity of marine animals with stage-resolved stratigraphic ranges^22^,^23^,^24^. We used epoch level resolution for the first four Cambrian stages, as many taxa from this interval are not accurately resolved to the stage level. Our data set is not biased by the inclusion of living taxa, as we have excluded the Holocene stage (0.117 Myr ago to the present) in this analysis, but including it has almost no influence on ecological diversity in the modern and does not qualitatively alter our conclusions (Supplementary Fig. 6). In addition, the proportion of missing taxa in our data does not vary dramatically as a function of time because the functional traits coded here are relatively straightforward to determine even for entirely extinct clades (Supplementary Fig. 7). If anything, incompleteness increases towards the present day. Moreover, many of the missing Cenozoic genera are molluscs, because gastropods and bivalves often exhibit variation in ecological mode at low taxonomic levels. These classes occupy many of the rare ecological modes and so coding them would tend to increase the trajectories of the rarefaction curves. Therefore, even if some of the missing Palaeozoic genera also occupied rare or otherwise unoccupied ecological modes, it appears unlikely that the offset in rarefaction curve trajectories among eras results simply from improved knowledge of ecological modes toward the present day and we suspect that our results are conservative. Furthermore, the fossil record most frequently preserves taxa with mineralized ‘hard-parts’, and only infrequently preserves soft-bodied taxa lacking skeletal elements. Using Sepkoski’s genus compendium^24^, Bambach *et al*.^14^ compared the differences in modes of life between recent taxa that are well represented in the fossil record and those that are not. They found that overall there was little bias in the modes of life occupied by soft-bodied genera and those occupied by animals with mineralized skeletons. Given that the same compendium^24^ also forms the foundation of our data set, the results presented here for skeletonized animals is also likely to be an unbiased sample of all marine animals.

### Ecological assignments

We assigned each genus in our database to an ecological mode of life^14^,^15^. Assignments for tiering position in relation to the sea floor, motility level and feeding strategy were taken from the literature and based on the first principles of functional morphology (all data and references are available online as Supplementary Data 1). All ecological assignments were based on the life habits of the adult form. In the exceedingly rare cases where a genus occupied more than one ecological category for a given axis as an adult, the single ecological category that describes the majority of species in the genus was chosen.

### Statistical analyses

All statistical analyses were performed in either the R computer-programming environment^34^ or in Python^35^. The 95% confidence intervals for the expected number of ecological modes of life filled in each stage (Fig. 3) was generated by tabulating the total number of modes of life occupied and the actual number of genera that fill these ecological modes across the Phanerozoic. A times-series was created by randomly drawing ecological modes from a pool consisting of the frequencies of each mode across all geologic time. In each stage, *X* draws were made from this pool, where *X* was the actual number of genera in that geologic stage. This process was repeated 10,000 times to generate the 95% confidence interval for the expected number of modes of life occupied given the standing genus diversity in each stage. This 95% confidence interval is the prediction for the time-constant model (Fig. 1b), where there is a single relationship between taxonomic diversity (number of genera) and ecological modes across the Phanerozoic. This analysis was also done weighting each stage identically regardless of the actual number of genera in the stage (Supplementary Fig. 8) to avoid biasing early low diversity stages towards higher expected number of ecological modes (due to more recent stages with much higher genus diversity) and by utilizing only the Holocene number of modes of life and number of genera to create the 95% confidence interval across the Phanerozoic (Supplementary Fig. 9). These alternative approaches make little difference to the expectation of number of modes in each geologic stage and do not alter any conclusions of this work.

To assess whether ecological modes with high taxonomic diversity preferentially lost diversity across era-bounding mass extinctions we regressed the standardized difference in diversity pre- and post-extinction event, with the taxonomic diversity pre-extinction event. Specifically, we used 262.5 to 251.7 Myr ago at the P/Tr (as our data clearly show decline in taxonomic diversity and ecological modes before the 252 Myr ago boundary; Fig. 3) and from 69.05 to 63 Myr ago for the K/Pg (see also Fig. 3). In both cases, this allows for comparison from a pre-extinction baseline to a post-extinction, but pre-recovery interval.

To examine whether early functional differentiation within classes could predict later taxonomic diversity patterns we determined the correlation between the number of modes of life within classes at the end of the Ordovician (Katian stage: 445 Myr ago) with the taxonomic diversity in the Pleistocene (as a surrogate for modern taxonomic diversity, as Holocene taxonomic diversity values are strongly positively influenced by living taxa). We also determined the correlation between the number of modes of life within classes at the end of the Ordovician (Katian stage: 445 Myr ago) with taxonomic diversity at that time and across the entire Phanerozoic.

To determine whether correlation between the number of genera in a class and the number of modes of life in a class increased over the course of evolutionary history we calculated the Pearson’s correlation coefficient during all geologic stages. For each stage, all classes with fewer than 10 genera during that stage were excluded from the analysis, as classes with low diversity do not have sufficient sample sizes to reliably estimate number of ecological modes. Stages with fewer than three classes remaining after this cut off were excluded from downstream analysis (most of these occurred in the Cambrian), as a correlation coefficient cannot be reliably calculated with only three values. Number of genera and number of ecological modes were both log transformed before calculating correlation coefficients. To examine trends across geological time we tested whether the estimated correlation coefficient differed between the Palaeozoic, the Mesozoic and the Cenozoic using a generalized least-squares model with a fixed effect of era and a temporal autocorrelation structure. The correlation in residuals between two time points was best described by an exponential decay function based on the amount of time between the two samples. We used a likelihood ratio test to examine whether era significantly explained differences in the data by comparing the full model with one that had an exponential autocorrelation structure but without the effect of era.

## Author contributions

M.L.K., N.A.H. and J.L.P. designed the study and collected the data. M.L.K., L.O.F., N.A.H. and J.L.P. analysed the data. M.L.K., N.A.H., L.O.F. and J.L.P. interpreted the data and wrote the paper.

## Additional information

**How to cite this article:** Knope, M. L. *et al*. Limited role of functional differentiation in early diversification of animals. *Nat. Commun.* 6:6455 doi: 10.1038/ncomms7455 (2015).

## Supplementary Material

Supplementary Information Supplementary Figures 1-9

Supplementary Dataset 1 The Supplemental Data File includes the phylum and class of each fossil genus (taxon_name), the age of first appearance of that genus in the fossil record (in millions of years ago; fad_age), the stage of first appearance (fad_int), the age of last appearance (lad_age), the stage of last appearance (lad_int), and numeric codes for habitat tiering (tiering), motility (motility), feeding mode (feeding), and their combination (ecological mode) following the numbering system of Bambach et al. (2007), and the references used to obtain the ecological modes (eco_ref).
