# Supplementary material for: Limited role of functional differentiation in early diversification of animals
Source: Nat Commun. 2015 Mar 4;6:6455. doi: 10.1038/ncomms7455 (PMC4366486; doi:10.1038/ncomms7455)
Supplement: Supplementary Information — Supplementary Figures 1-9 [file ncomms7455-s1.pdf]

Supplementary figures

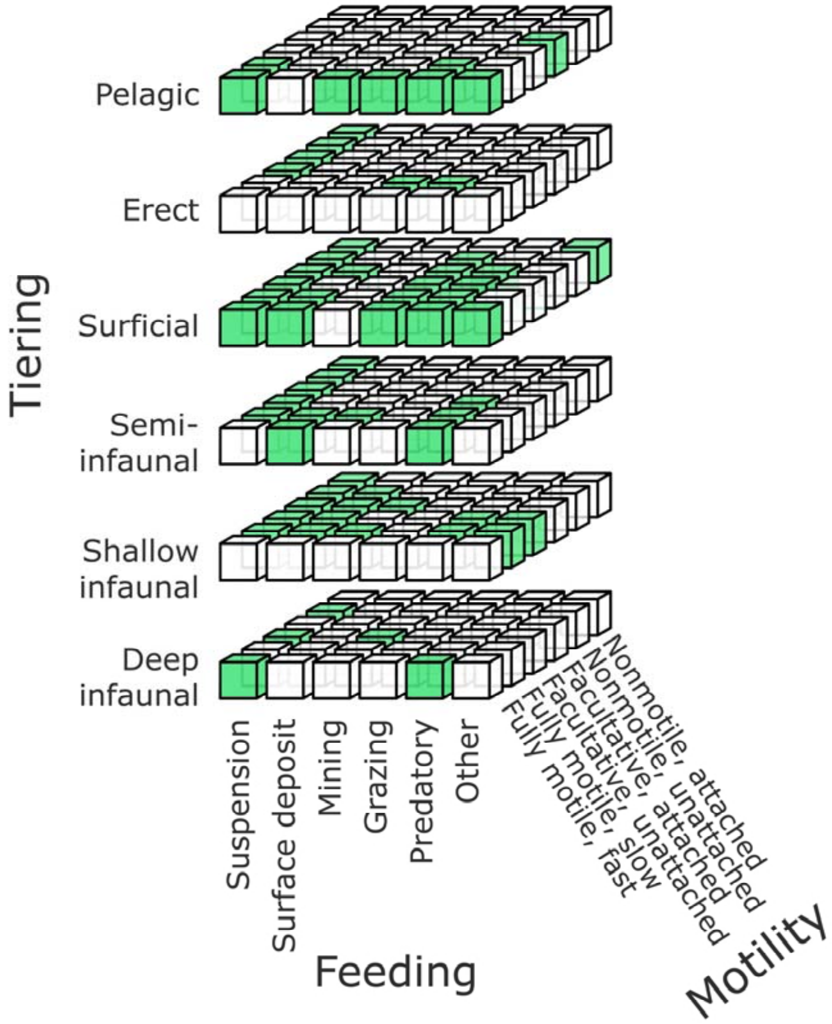

Supplementary Figure 1. Ecospace occupation of marine animals across the Phanerozoic. Across the Phanerozoic (541 Ma – present) fossil marine animals with stage resolved stratigraphic ranges have occupied 65 ecological modes of life.

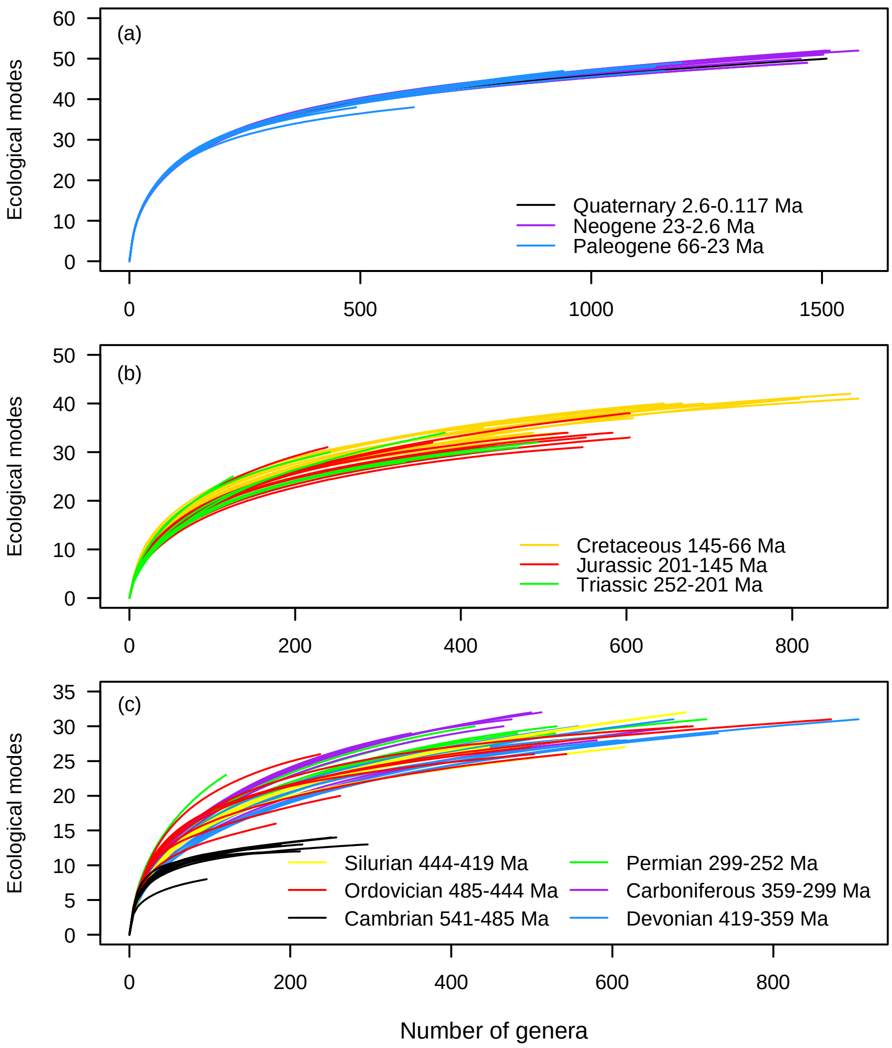

Supplementary Figure 2. Rarefaction curves for each geologic era with stages color-coded by the geologic period. Individual rarefaction curves are for each geologic stage color coded by the geologic period they belong to. (a) Rarefaction curves for stages within the Cenozoic have similar trajectories regardless of geologic period, although taxonomic diversity is lower in the Paleogene. (b) Rarefaction curves for stages within the Mesozoic also largely overlap, although some Cretaceous stages not only have higher taxonomic diversity but also occupy more modes of life. (c) Rarefaction curves for stages within the Paleozoic largely overlap; however, the Cambrian period displays lower filling of ecological modes. Note that x-axis and y-axis scales differ for each geologic era.

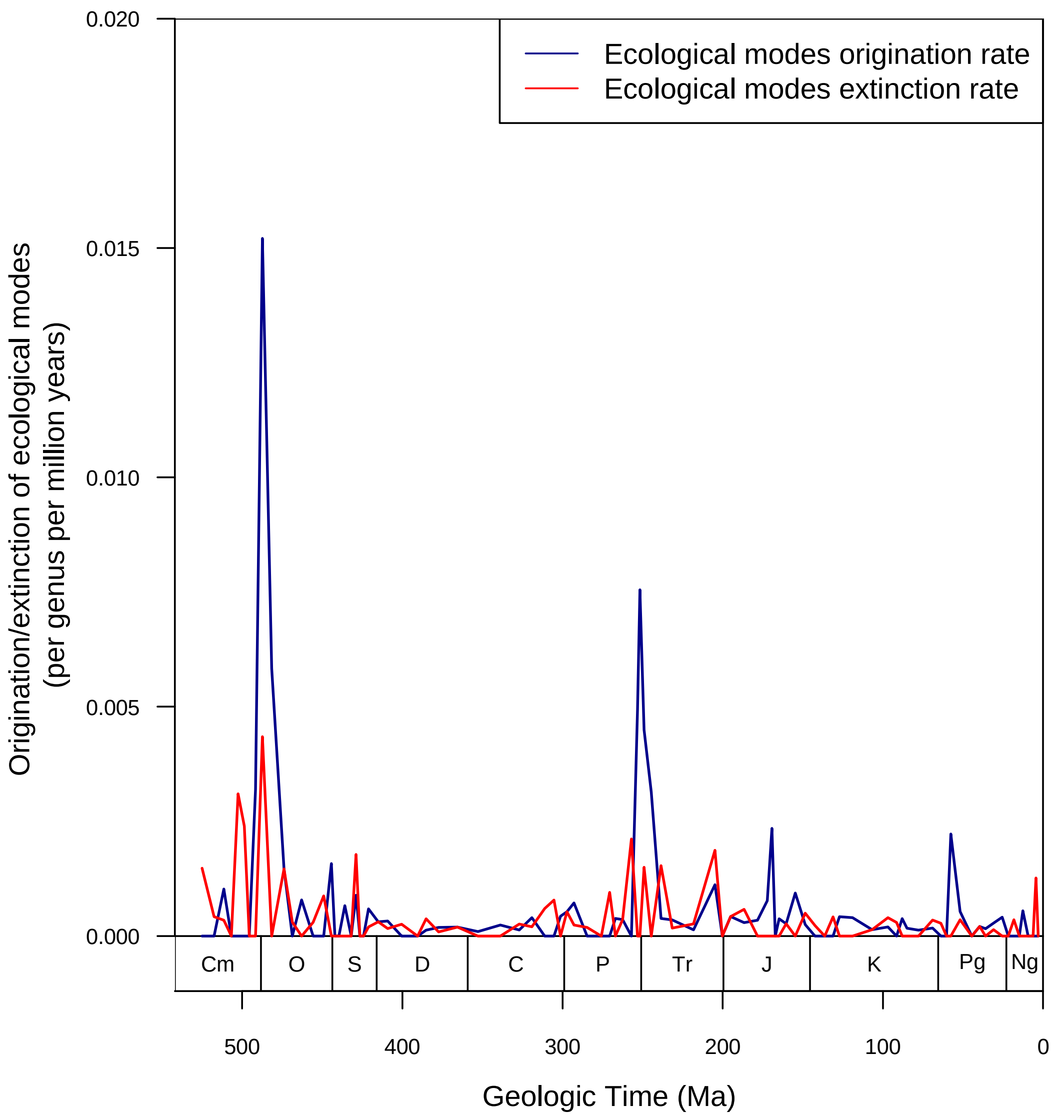

Supplementary Figure 3. Rate of origination and extinction of ecological modes of life detected. Rates of origination and extinction of ecological modes were calculated on a per genus per million years basis. Note the greatest rates of origination of ecological modes are during the Ordovician period and in the recovery interval following the era-bounding mass extinctions (P/Tr at 252 Ma and K/Pg at 66 Ma).

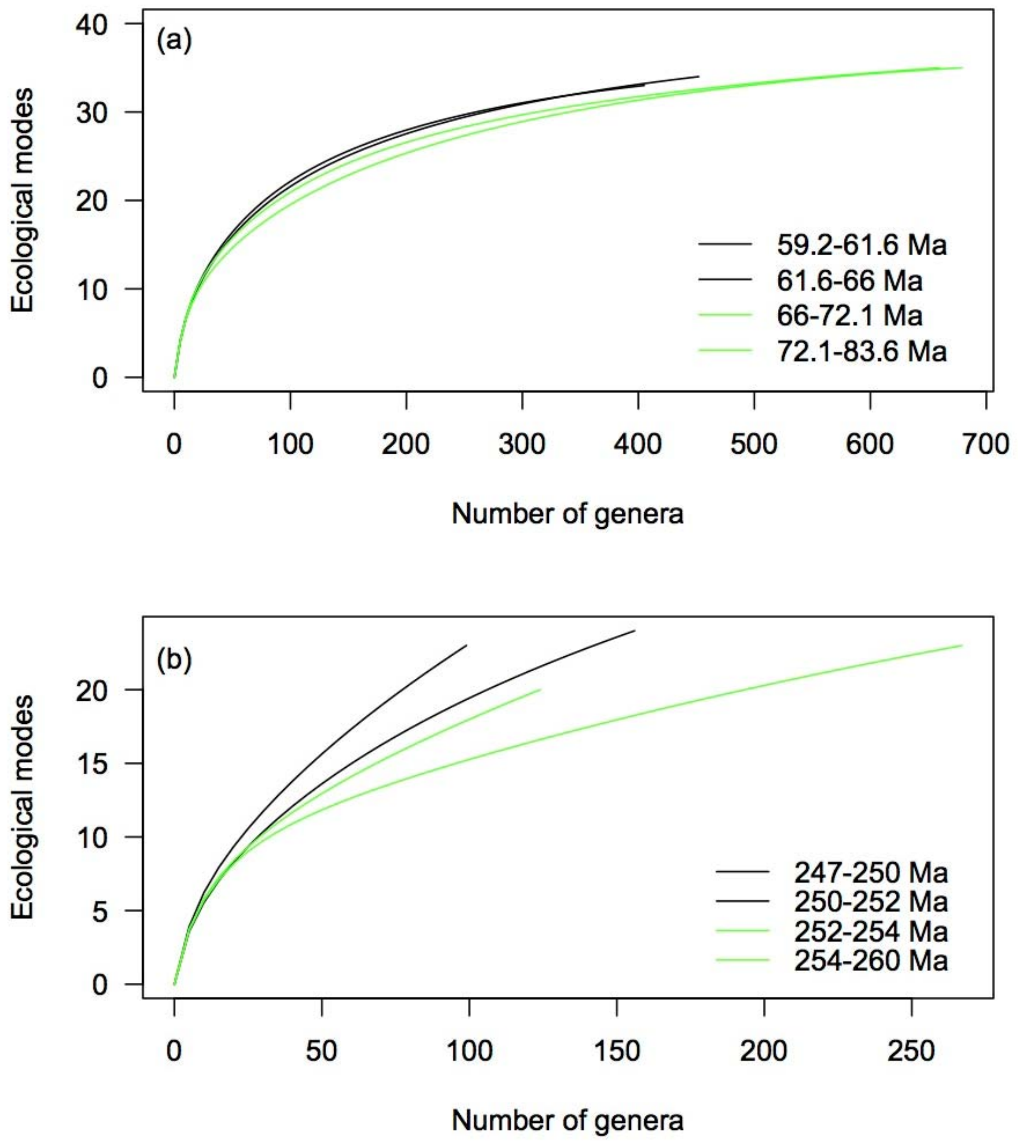

Supplementary Figure 4. Rarefaction curves at mass extinctions (two stages before in green and two stages after in black). (a) Cretaceous-Paleogene extinction event. Rarefaction curves by geologic stage. Stages in legend are organized by the most recent stage (Selandian [59.2-61.5 Ma]) at top and oldest stage (Campanian [72.1-83.6 Ma]) at bottom. In both cases, while taxonomic diversity is greatly reduced after the extinction event, ecological modes remain at a high level indicating that taxonomic diversity is preferentially lost within previously well-filled ecological modes. (b) Permian-Triassic extinction event. Stages in legend are organized by the most recent stage (Olenekian [247-250 Ma]) at top and oldest stage (Wuchiapingian [254-260 Ma]) at bottom.

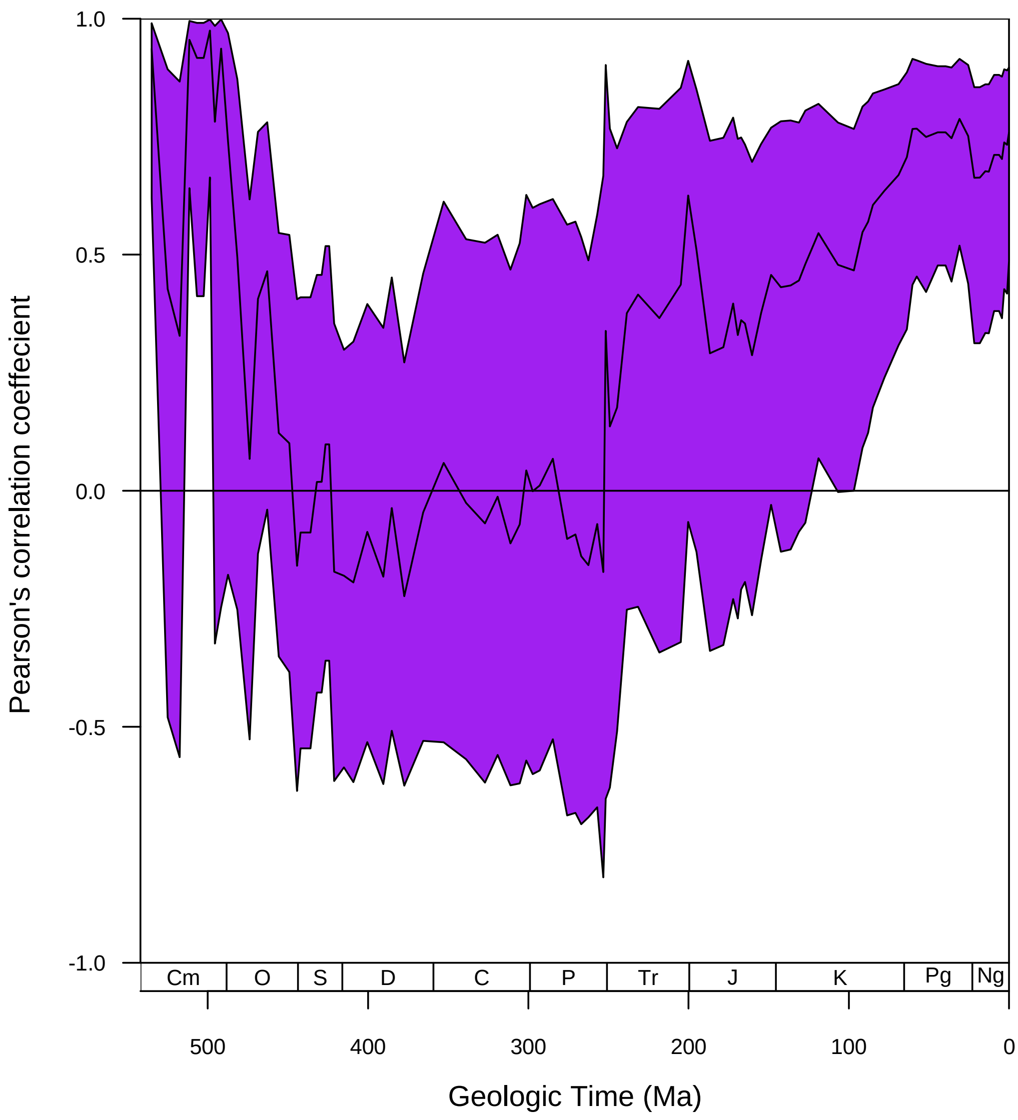

Supplementary Figure 5. Increased correlation between taxonomic diversity and ecological modes within marine animal classes across time. Pearson's correlation coefficient values for ecological modes of life and genus diversity within Linnaean classes. Purple envelope indicates the 95% confidence interval generated by bootstrapping; where both upper and lower bound become positive, indicates statistically significant positive relationship ($p < 0.05$) between ecology and diversity. Correlations are high in the Cambrian, but statistical confidence is low due to small numbers of genera and modes of life.

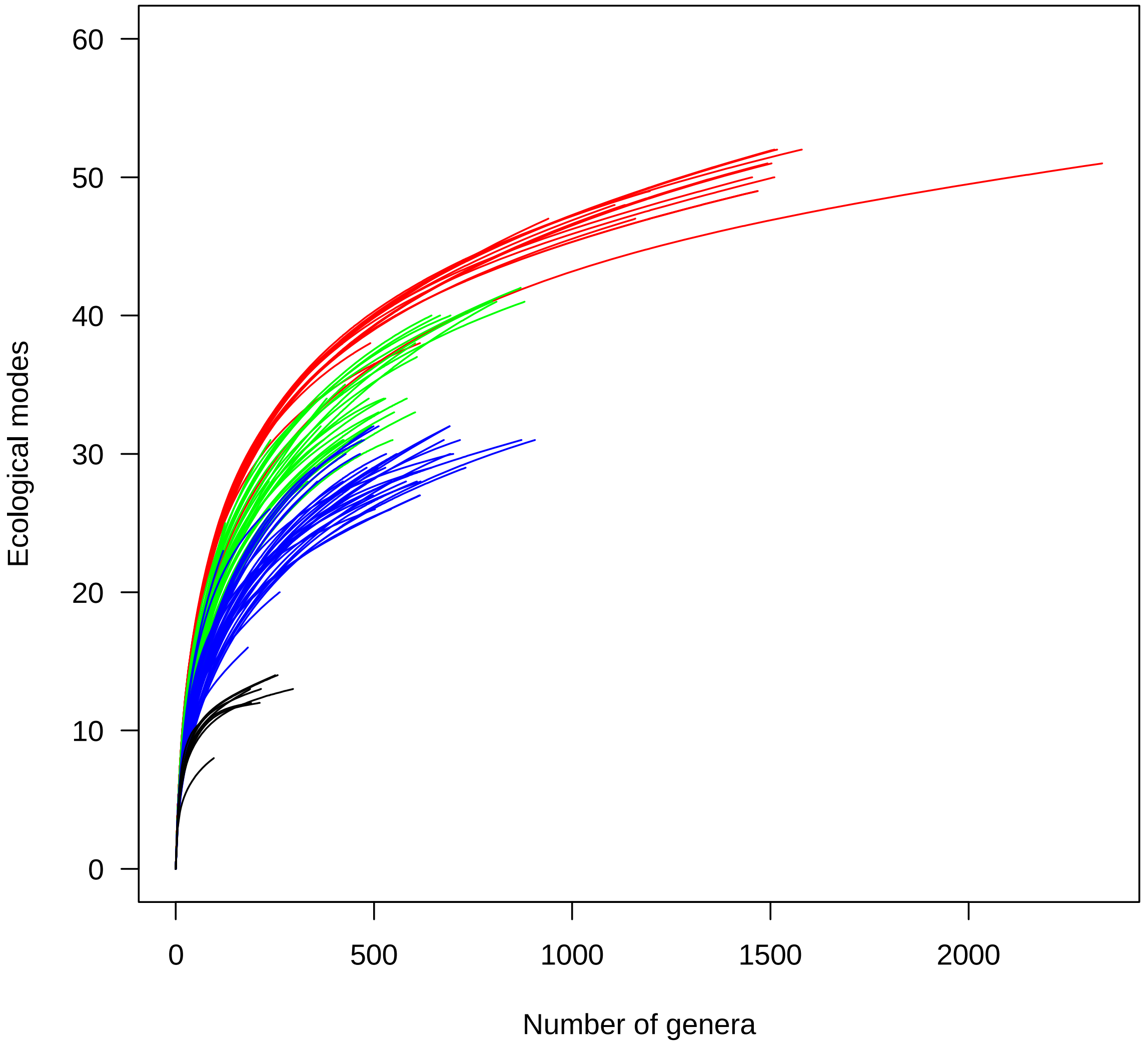

Supplementary Figure 6. Rarefaction curves of relationship between genus diversity and ecological modes of life across all geologic stages including the Holocene. Note that inclusion of the Holocene (single red rarefaction curve that extends far beyond the others) increases genus diversity but not total ecological modes.

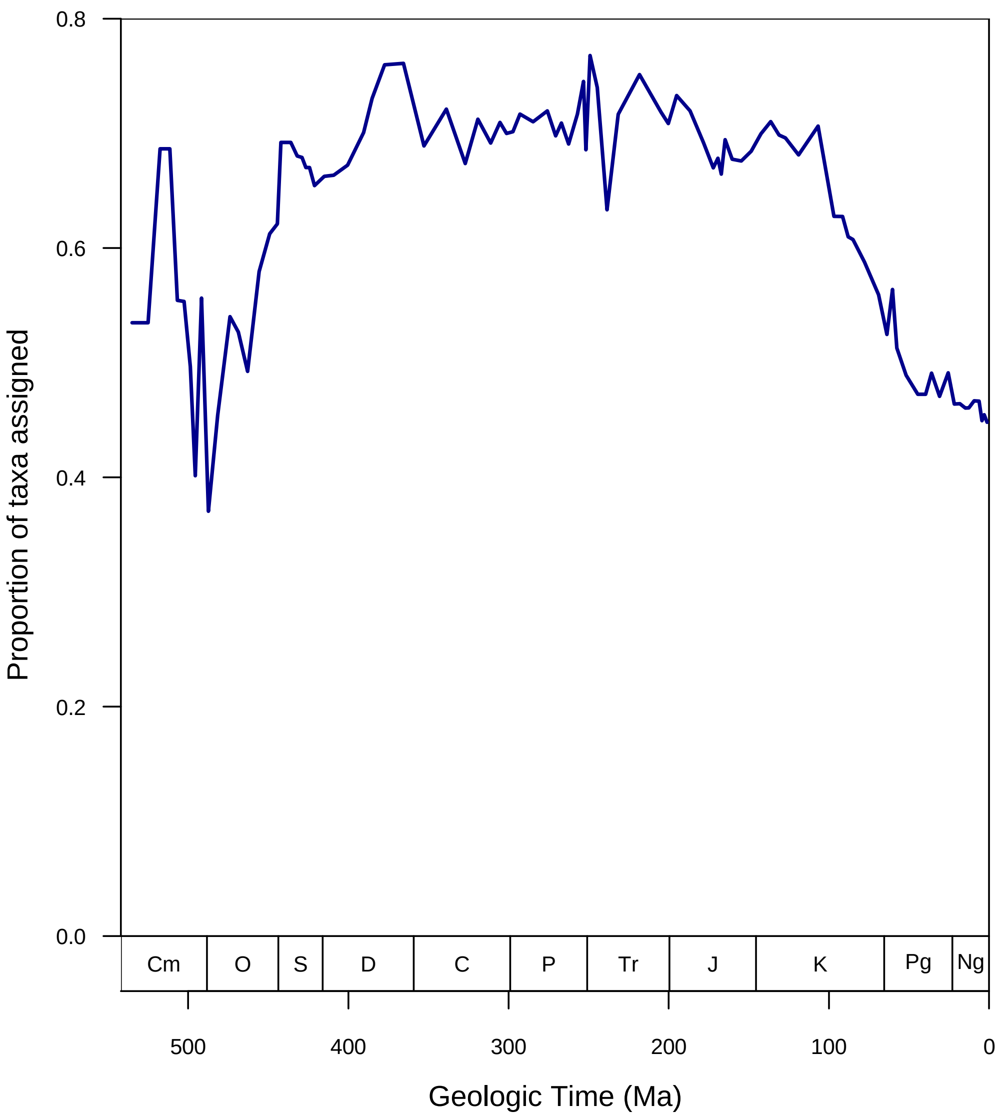

Supplementary Figure 7. Coverage of the fossil record with ecological assignments. Proportion of marine animal genera with stage resolved stratigraphic ranges that we assigned to an ecological mode in each stage across the Phanerozoic.

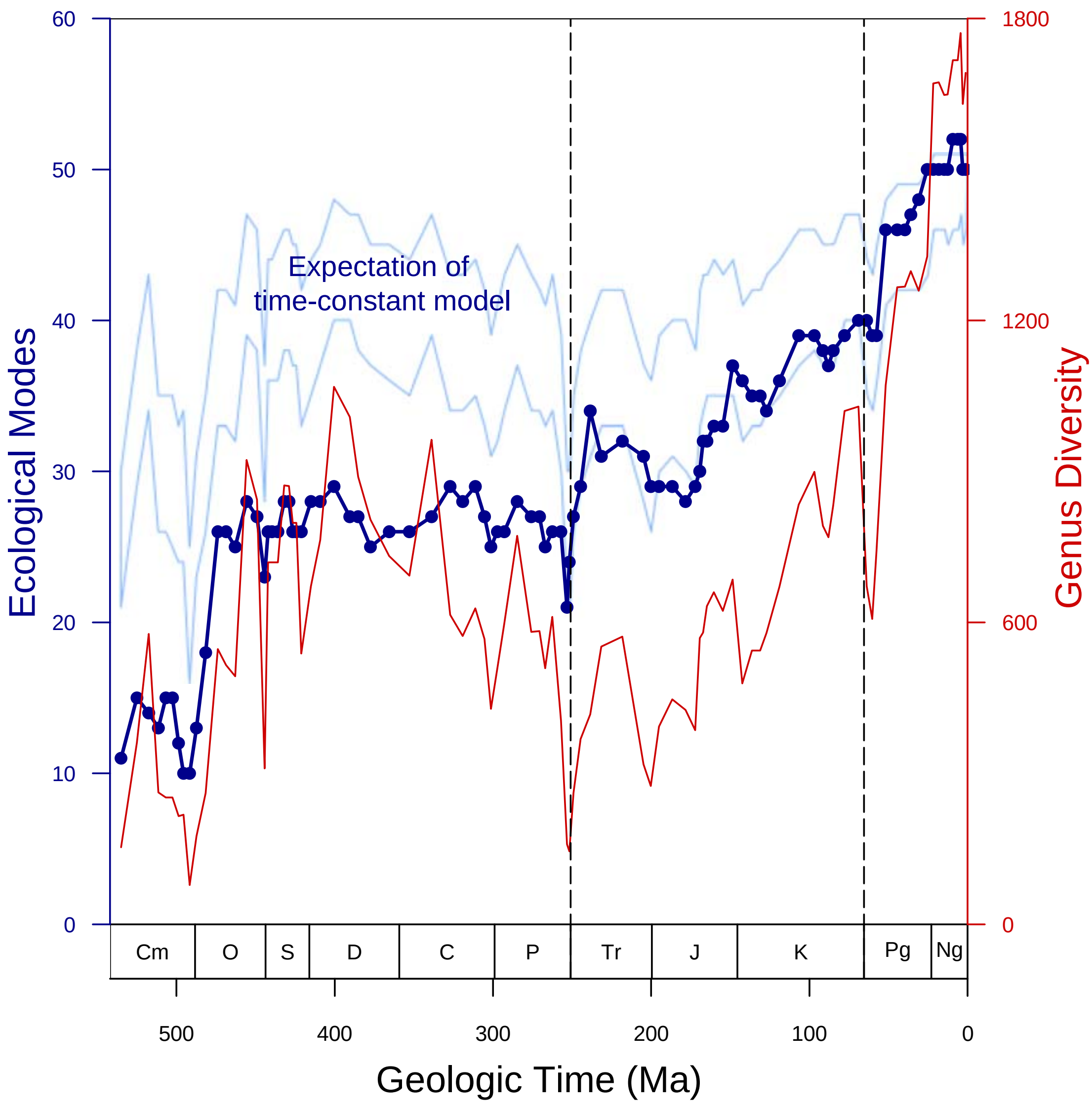

Supplementary Figure 8. Changes in the number of ecological modes of life occupied by marine animals over the past 541 million years, as compared to a time-constant model based on the Holocene. Heavy blue line with filled points depicts ecological modes; thin red line depicts genus diversity in our study with an assignment to an ecological mode; the blue polygon illustrates the 95% confidence interval for the expected number of ecological modes relative to observed genus diversity in each stage under the time-constant scenario (Fig. 1b) where the expectation of the time-constant model is based on the frequencies of ecological modes filled by genera in the Holocene only. Dashed vertical lines demarcate the era-bounding mass extinctions at the P/Tr (252 Ma) and K/Pg (66 Ma) extinction horizons.

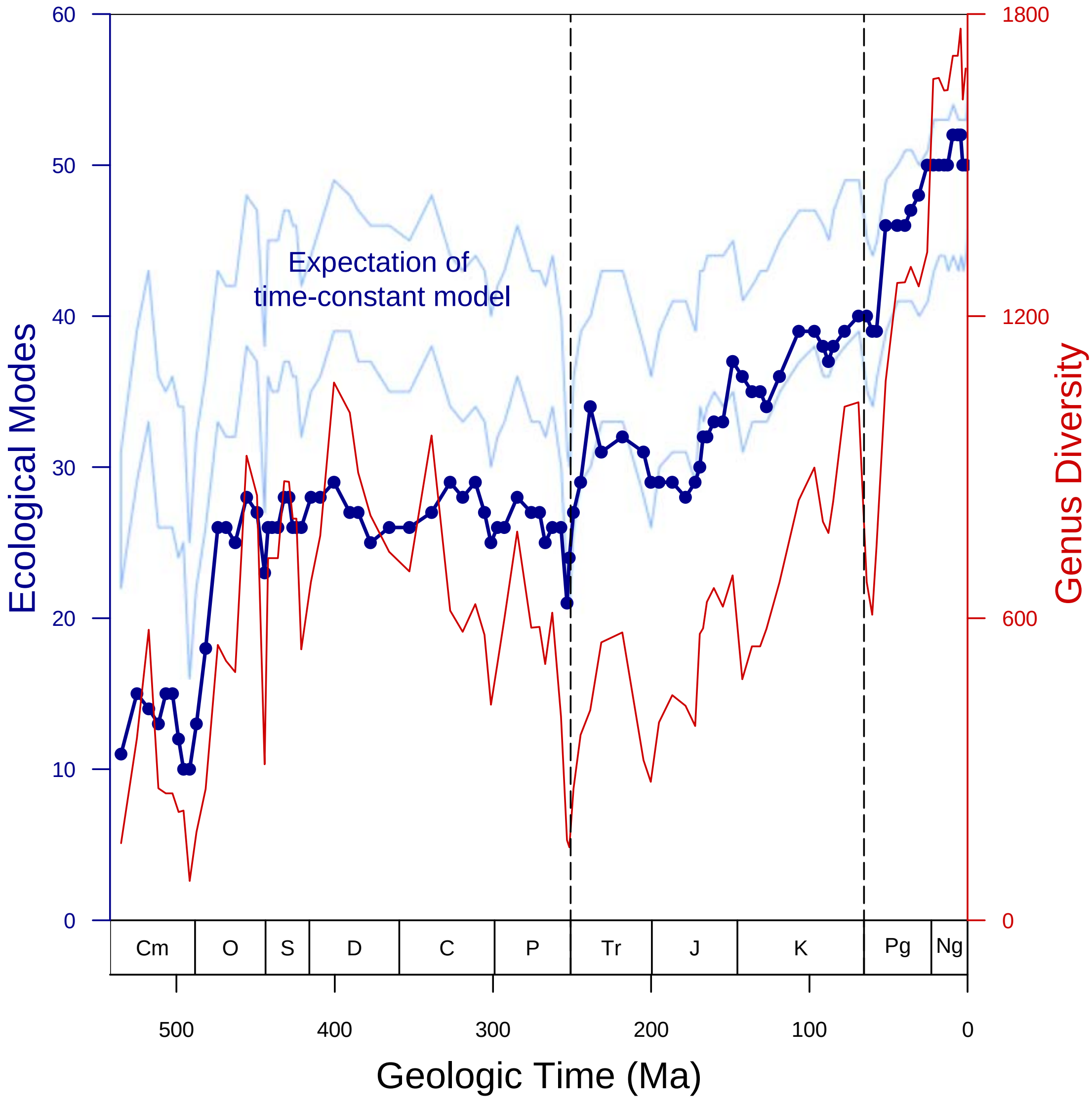

Supplementary Figure 9. Changes in the number of ecological modes of life occupied by marine animals over the past 541 million years, as compared to a time-constant model based on all Phanerozoic stages weighted equally. Heavy blue line with filled points depicts ecological modes; thin red line depicts genus diversity in our study with an assignment to an ecological mode; the blue polygon illustrates the 95% confidence interval for the expected number of ecological modes relative to observed genus diversity in each stage under the time-constant scenario (Fig. 1b) where expectation of the time-constant model is based on the frequencies of ecological modes filled by genus diversity across all stages in the Phanerozoic, with each stage given equal weight, regardless of its genus diversity. Dashed vertical lines demarcate the era-bounding mass extinctions at the P/Tr (252 Ma) and K/Pg (66 Ma) extinction horizons.
